# Safety and efficacy of outpatient bronchoscopy in lung transplant recipients - a single centre analysis of 3,197 procedures

**DOI:** 10.1186/2047-1440-3-11

**Published:** 2014-05-27

**Authors:** Jessica Rademacher, Hendrik Suhling, Mark Greer, Axel Haverich, Tobias Welte, Gregor Warnecke, Jens Gottlieb

**Affiliations:** 1 Department of Respiratory Medicine, Hannover Medical School, Carl Neuberg Str. 1, 30625 Hannover, Germany; 2 Department of Cardiothoracic, Transplantation and Vascular Surgery, Hannover Medical School, Hannover, Germany

**Keywords:** Bronchoscopy, Complications, Lung transplantation, Pneumothorax

## Abstract

**Background:**

Bronchoscopy represents an important diagnostic and therapeutic tool in the management of lung transplant (LTx) recipients. Outpatient bronchoscopy reduces health costs and may improve quality of life amongst these patients. This retrospective study assessed the safety and efficacy of outpatient bronchoscopy including trans-bronchial biopsy.

**Methods:**

All outpatient bronchoscopies performed on lung transplant recipients between 1 August 2008 and 31 January 2011 were reviewed. Sample quality, duration and complications were recorded. Cost analysis was performed from local trust financial data.

**Results:**

A total of 3,197 bronchoscopies were performed on 571 LTx recipients under topical anaesthesia. Fourteen percent of examinations required intravenous sedation. In 79.8% of examinations no complications were observed. Most complications were minor (17.9%) including cough (5.3%) and minimal bleeding after trans-bronchial biopsy (7.8%). Major complications (2.3%) were pneumothorax, severe bleeding and severe desaturation. No attributable deaths were recorded during the observation period. Quality of examination based on bronchoalveolar lavage recovery median (>50%) and biopsy results was adequate at 75% and 77.4%, respectively. Independent risk factors associated with complication were long-term oxygen therapy, sedation before examination, balloon dilatation and transbronchial biopsy. After excluding high-risk procedures annual savings per patient (2.2 bronchoscopies per year) were 2140€.

**Conclusions:**

Outpatient bronchoscopy after LTx is safe. The low complication rate could be attributed to withholding of intravenous sedation. Furthermore, it reduces health community costs.

## Background

Fibre-optic bronchoscopy has remained the gold standard in managing tracheobronchial pathology since its introduction in 1968 [1-4]. It continues to play an indispensable role in the follow-up care of lung transplant recipients, allowing direct visual assessment of the airways and facilitating diagnostic sampling from the lower respiratory tract. It is the minimum prerequisite for performing transbronchial biopsies (TBBs), which since the initial reports in 1988 [5] have become an established diagnostic tool in managing suspected acute rejection or infection in lung transplant recipients [6]. Interventional fibre-optic bronchoscopy is also gaining prominence in the management of post-transplant airway complications. Although theoretically better tolerated due to organ denervation, previous studies have reported on increased bleeding risk independent of coagulation status, platelet count or aspirin use amongst lung transplant recipients [7].

At present, the major obstacles to long-term success following lung transplantation remain infection and chronic lung allograft dysfunction (CLAD). Other common problems are acute rejection, airway complications and primary graft dysfunction [8]. Flexible bronchoscopy, therefore, represents an essential diagnostic and therapeutic tool. Bronchoalveolar lavage (BAL) remains key in diagnosing infection [9,10] and routine TBB permits the early detection of acute rejection, facilitating both early initiation of treatment as well as follow-up sampling to ensure successful resolution or to determine the need for further treatment [11]. Previous studies have demonstrated the safety of fibre-optic bronchoscopy as an outpatient procedure [12,13], although no such reports involving lung transplant (LTx) cohorts exist.

In an attempt to simultaneously improve quality of life in transplant recipients and reduce health costs, many centres have adopted performing bronchoscopy on an outpatient basis. As limited data exist regarding the efficacy and safety of this approach, we analysed data and results of all bronchoscopies at our centre to profile these aspects amongst LTx recipients on an outpatient basis.

## Methods

Patients: All outpatient bronchoscopies in LTx recipients performed between 1 August 2008 and 31 January 2011 at the Hannover Medical School (MHH) were reviewed. The MHH is a tertiary referral centre specialising in lung transplantation, currently performing more than 120 lung and heart-lung transplantations annually. At present, more than 750 patients regularly attend for outpatient follow-up.

Procedures: As part of our surveillance program, outpatient bronchoscopy is performed at 1, 3, 6, 12, 18, 24 and 30 months post-transplantation and surveillance biopsies are performed at 1, 3, 6 and 12 months to assess for occult rejection. Prior to bronchoscopy, all patients undergo clinical assessment, blood testing, blood gas analysis, spirometry and chest x-ray. In addition to routine bronchoscopy, further procedures are performed to investigate suspected infection or unclear deteriorations in graft function. Contraindications for bronchoscopy in LTx outpatients are refractory hypoxaemia (oxygen saturation <90% under 6 litre oxygen supply), pCO2 > 55 mmHg and haemodynamic instability.

All procedures were performed using flexible videoscopes (Olympus, Tokyo, Japan; Type P180, Q180, T180) and were recorded. During intervention patients received nasal oxygen, at least 2 L/minute to maintain SaO_2_ > 92%. Nasal and oro-pharyngeal anaesthesia was performed with 8 ml nebulised 2% lidocaine followed by 2% lidocaine instilled through the bronchoscope directly onto the vocal cords and proximal airways (total volume approximately 10 to 15 ml). Intravenously administered midazolam (2 to 5 mg) was used for sedation as required. Heart rate and oxygen saturation were monitored via pulse oximetry throughout the procedure. In cases of severe coughing, anti-tussive medication (morphine 2.5 to 5 mg s.c.) was used.

**Figure 1:**
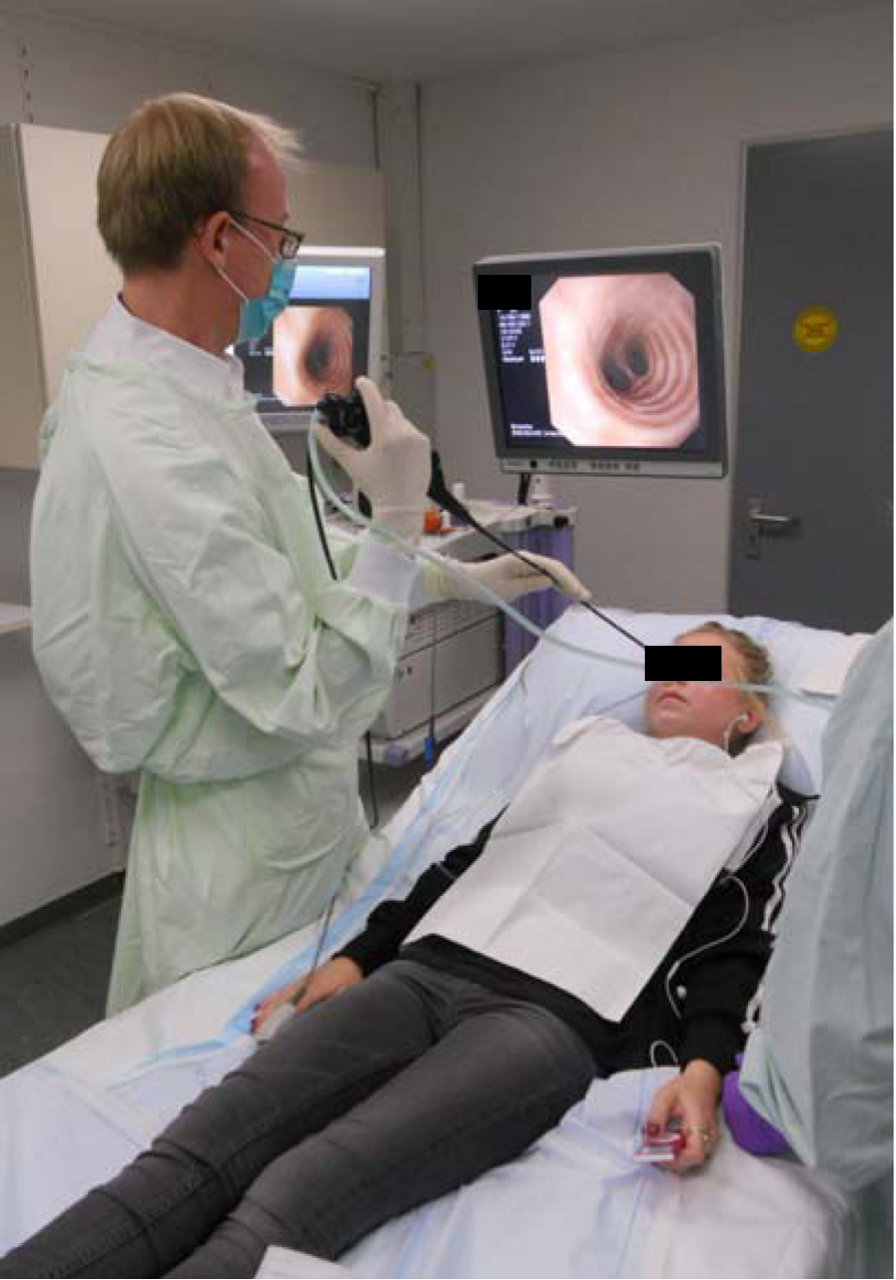
This figure shows a bronchoscopy in a non- sedated LTx outpatient (22 years old, cystic fibrosis), listening to self-selected music.

Patient position was carefully chosen with the patient being in a semi-recumbent position and the doctor by the side of the patient allowing permanent visual eye-to eye contact to the patient. All patients received training in relaxation methods during bronchoscopy in the early post-operative phase by bronchoscopy staff (nurse and doctor). Methods include bio feedback, for example, breathing exercises, visual imagery, muscle relaxation exercise plus simple hand holding and talking down strategies. Inspection of the central airways was performed as recommended followed by BAL with 6 × 20 ml aliquots of 0.9% saline instilled and gently aspirated from a sub-segmental bronchus in the region of interest, middle lobe or lingula. TBB was performed without fluoroscopy in a single lobe, involving sampling from several segments with the goal of obtaining five tissue samples as recommended [14]. Bleeding post-biopsy was managed by repeatedly instilling aliquots of ice-cold NaCl or epinephrine (1:20.000) into the bleeding source through the wedged bronchoscope. Figure 1 illustrates our outpatient bronchoscopy setting involving a young cystic fibrosis patient without sedation.

Definitions: Each BAL sample underwent microbiological and virological (immunofluorescence) testing. BAL fluids were submitted to direct immunofluorescence testing (IFT) specifically for influenza virus A and B, parainfluenza virus I–III, respiratory-syncytial virus, human metapneumovirus and adenovirus. Reverse transcriptase real-time PCR was performed for all patients with a negative IFT and a strong clinical suspicion of viral infection. TBBs were reviewed by an experienced pathologist and graded according to the International Society of Heart & Lung Transplantation (ISHLT) criteria for pulmonary allograft rejection. Sample quality and reported complications were recorded and entered into a database. Sampling adequacy was defined as a BAL recovery rate >50% and TBBs allowing categorical A (acute rejection) and B (lymphocytic bronchiolitis) ISHLT staging. Procedure duration was recorded from scope insertion to its subsequent withdrawal from the patient. Post-procedure observation lasted for one hour following standard bronchoscopy, with patients undergoing TBB being monitored for two hours, including repeat x-ray to exclude pneumothorax before discharge. Clinicians performing the procedure were classified into three groups based on experience: junior resident <3 years, senior resident 4 to 6 years and fellow. TBB, balloon dilatation, argon therapy and mitomycin application were defined *a priori* as high-risk procedures. Serious complications were defined as death, hospitalization, pneumothorax, severe bleeding and protracted hypoxia (SaO2 < 92%) after the procedure. Bleeding severity was assessed subjectively by individual clinicians as mild, moderate or severe. Non-severe complications were defined as minor.

Costs were calculated based on the trust local hospital financial data. The costs for outpatient bronchoscopy took into account the cost of the equipment, running and staff costs. Inpatient costing was geared to the diagnosis related groups (DRG) - system in Germany. The theoretical number of procedures per year was calculated by extrapolation of the average number per year performed in the study period.

This study was performed with the approval of the ethics committee of the Medical School of Hannover (Prof. Dr. H.D. Tröger; No. 1734–2013).

Statistical analysis: Numeric data are reported as median and interquartile ranges ((IQR) 25% and 75%). All reported *P* values are two sided unless otherwise indicated. *P* values less than 0.05 were considered statistically significant. Categorical variables were analysed using chi-square tests. A multivariate analysis for complications was performed using stepwise logistic regression with hazard ratios being calculated.

## Results

During the observation period, a total of 3,197 outpatient bronchoscopies were performed on 571 LTx recipients. The median patient age was 47.5 years (54.5% male) with a median of 652 days (IQR 37 to 1,952) post transplantation. Each patient underwent a median of four bronchoscopies (IQR 2 to 7). Patient demographics are displayed in Table 1. With regard to bronchoscopy indication, 27% were surveillance investigations, 18% post-intervention controls and 54% indicated due to deterioration in graft function. The most common diagnosis in indication bronchoscopies was bronchiolitis obliterans syndrome with declining graft function (38%). Nine percent of patients were oxygen-dependent prior to bronchoscopy. Fellows performed the majority of bronchoscopies at 57.1% (junior residents 18.5%, senior residents 24.4%).

**Table 1:** Patient characteristics

**Characteristic**	
Mean age, year (IQR)	47.5 (37.7 to 58.3)
Sex, M/F (%)	54.5/45.5
Underlying diseases (%)	
- Emphysema	34.4
- Idiopathic pulmonary fibrosis	25
- Cystic fibrosis	25.7
- Pulmonary arterial hypertension	7.8
- Other	7
Procedure (%)	
- Single lung transplantation	7.7
- Double lung transplantation	86.9
- Heart lung transplantation	5.4
Bronchiolitis obliterans syndrome (%)	38.4
Oxygen dependence (%)	4.5
Number of procedures per patient (IQR)	4 (2 to 7)

IQR, interquartile range.

The investigations lasted a median 15 (IQR 10 to 20) minutes, with those involving interventions such as TBB, balloon dilatation, argon therapy or mitomycin application taking considerably longer (13 versus 19 minutes; *P* <0.05). Topical anaesthesia was administered in all bronchoscopies, anti-tussive treatment was needed in 4% and only 14% of examinations required intravenous sedation. Common interventions included BAL in 81.4% and TBB in 22.6%. Argon therapy (14.7%), balloon dilatation (3.8%) and mitomycin application (4%) were performed less frequently, reflecting the prevalence of common complications following transplantation. In examinations involving TBB, the median number of biopsies was five (IQR four to six). Histological review of the obtained tissue was sufficient for ISHLT A-grading in 96.2% and B-grading in 77.4%.

No complications were detected in 79.8% of the investigations (Table 2). High-risk procedures accounted for 428/647 of those with recorded complications. Serious complications arose in 2.3% of all bronchoscopies, although amongst high-risk procedures the rate was surprisingly lower (1.7%). Amongst senior residents, 286/781 (36.6%) procedures performed were high-risk, with a serious complication rate of 1.2%. Fellows carried out the most bronchoscopies, with 947/1,825 (51.9%) being high-risk, resulting in a higher serious complication rate (3.1%). Recorded complications were mostly minor (n = 572; 17.9%) and consisted mainly of excessive coughing and minimal bleeding following TBB. Three patients (0.1% of the total) suffered pneumothoraces after biopsy (0.4% of those with TBB), all of which were small and asymptomatic and did not require chest drain insertion. These patients were admitted for observation with x-ray control after 24 hours to confirm resolution. One episode of vasovagal syncope after discharge following bronchoscopy was reported, with the patient being observed as an inpatient for 24 hours. Overall, four patients needed hospitalization with one patient in the ICU. Predictors for any complications included intravenous sedation (*P* <0.001), long-term oxygen (*P* = 0.006), balloon dilatation (*P* <0.001) and TBB (*P* <0.001). No deaths and no mechanical ventilation within 72 hours of bronchoscopy were recorded.

**Table 2:** Complications observed in our analysis (multiple complications allowed)

**Complication**	**Percentage**
Any complication	20.2
Bleeding, absolute	10.2
• Bleeding, minimal	7.8
• Bleeding, moderate	2.3
• Bleeding, severe	0.1
Excessive coughing	5.3
Desaturation	2.2
Vomiting	1.4
Laryngospasm	0.9
Epistaxis	0.2
Pneumothorax	0.1
Other	0.1

The actual cost of a single outpatient bronchoscopy was calculated as 173€. In the case of hospitalisation, costs vary from 1,146 up to 3,031 depending on the presence (20%) or absence of rejection and length of stay (one or two days). After excluding high-risk procedures (need for sedation, balloon bronchoplasty and long-term oxygen) annual savings per patient (2.2 bronchoscopies per year) was 2,140€.

## Discussion

This study represents the largest retrospective analysis of the safety and efficacy of outpatient bronchoscopy in lung transplant recipients. Our findings emphasise good safety outcomes, with 80% of procedures being complication-free and with a low rate of serious complications (2.3%). Given the proposed increased risks of desaturation and bleeding in transplant cohorts and the number of high-risk interventions, these rates were lower than expected.

Existing data for complications associated with bronchoscopy in general have resulted predominantly from surveys and retrospective studies. Our complication rates appear broadly similar to those reported by Ouelette *et al.* in their supervised fellowship training program on inpatients, which demonstrated a complication rate of 2.1% in 3,538 procedures [15]. Credle *et al.* in a survey of 250 physicians, identified a major complication rate of 0.08% with regard to bronchoscopy [16], which correlated with the findings of Suratt *et al.* who surveyed 1,041 physicians performing bronchoscopy, that returned a complication rate of 0.3% [17]. A prospective analysis by Dreisin *et al.* consisting of 205 consecutive inpatient bronchoscopies, however, suggested much higher incidences of major or serious complication rates approaching 5% [18]. This discordance may be partly explained through variations in the definition of complications: major complications were similar, consisting of bronchospasm, laryngospasm, pneumothorax and hemoptysis. Minor complications occurred in 6%, but appeared much more comprehensive, including factors such as subsequent pulmonary infiltrates, dyspnea, maxillary sinusitis and hysterical reaction. The largest study to date by Jin *et al.* retrospectively reviewed 23,862 patients who underwent bronchoscopy [19]. They demonstrated a severe complication rate comparable to other recent analyses of 0.637%, consisting mainly of laryngo- and bronchospasm.

Data specific to bronchoscopy in lung transplant recipients is similarly limited, but figures available suggest broadly similar complication rates to non-transplanted patients [6]. Discordance however exists, with a study by McWilliams *et al.* reporting on complication rates in excess of 24% in patients beyond their first year after transplantation [20]. Interestingly, their cohort received substantially greater sedation, with patients receiving between 1 to 10 mg midazolam along with up to 200 mg propofol as required. By contrast Hopkins *et al*. reported much lower overall complication rates (6.35%) in a review of 1,235 bronchoscopies, consisting mainly of bleeding >100 ml (4%) and pneumothorax (1.46%). Again, all patients were fully sedated, this time with fentanyl and midazolam, and the procedure performed in an operating theatre environment [21]. A clear explanation for the difference in complication rates between these studies remains elusive. Regarding specific complications, we demonstrated comparable pneumothorax rates (0.1%) to earlier publications that ranged between 0 to 2% [21-25]. Bronchoscopy-associated mortality has been consistently reported as low, ranging between 0.01% to 0.04% [26,27], with Jin *et al.* reporting 0.013% [18] and Hopkins *et al.* recording no deaths [21]. As demonstrated by Surratt *et al*., underlying comorbidity, such as cardiovascular disease, pneumonia and cancer, appears most relevant in assessing mortality risk [17], underlying the need for careful cardiovascular and pulmonary assessment prior to bronchoscopy.

With regards to TBB-associated bleeding, a previous study by Chhajed *et al.* analysed several suspected risk factors amongst lung transplant recipients [28]. These included gender, transplant type, acute rejection, bronchiolitis obliterans syndrome (BOS) status, infection, number of biopsies taken, serum creatinine and time after transplantation. None of these factors proved significant. In comparison to general pulmonary patients, however, LTx recipients tend to be younger with lower comorbidity rates. In our study, intravenous sedation, oxygen dependency and specific interventions such as TBB and balloon dilatation were found to be significant risk factors for complications (Table 3). With regard to sedation, numerous studies support our findings [20,21,29-31] and it should be remembered that in our cohort,sedation was generally reserved for difficult, longer procedures that carried additional independent risks for complications. Our decision to withhold routine sedation is vindicated, both in terms of the low complication rates and on the findings of Colt and Morris. In a retrospective study they demonstrated improved peri-interventional feedback from patients and earlier return to pre-bronchoscopy functional state when sedation was withheld [32].

**Table 3:** Univariate analysis and multivariate analysis comparing serious and no serious complication was performed using forward regression analysis

	**Univariate analysis, chi-square**			**Multivariate analysis**	
**Variable**	**Serious complications, number (%)**	**Non serious complications number (%)**	***P*** **Value**	**Hazard ratio (95% CI)**	***P*** **value**
Number	74 (2.3)	3,092 (97.6)			
Resident niveau, yes	19 (25.3)	1353 (434)	0.002		0.069
Oxygen, yes	9 (12)	136 (4.4)	0.006	2.58 (1.21; 5.13)	0.013
Acute indication, yes	58 (77.3)	2,016 (64.6)	0.027		0.78
Age >60 years, yes	16 (21.3)	554 (17.8)	0.25		0.32
Age <25 years, yes	9 (12)	211 (6.8)	0.09		0.09
Time >20 min, yes	30 (40)	956 (30.6)	0.09		0.27
Bronchiolitis obliterans syndrome, yes	36 (48)	1,191 (38.2)	0.09		0.15
Balloon, yes	14 (18.9)	106 (3.4)	<0.001	5.8 (3.0; 11.5)	< 0.001
TBB, yes	3 (4.1)	718 (23.2)	<0.001	5.85 (1.827; 18.770)	0.003
BAL, yes	51 (68.9)	2,551 (82.2)	0.006		0.8
Argon therapy, yes	18 (24.3)	452 (14.6)	0.03		0.44
Sedation, yes	23 (30.7)	436 (14)	<0.001	2.12 (1.2; 3.6)	0.005
Mitomycin. yes	2 (2.7)	126 (4.1)	0.76	4.82 (1.064; 21.853)	0.041
Anti-tussive, yes	0	10 (0.3)	1		

All *P* values are two sided. BAL, bronchoalveolar lavage; CI, confidence interval; TBB, transbroncheal biopsy.

Patient preparation and coaching are important for performing bronchoscopy without sedation. Dubois *et al*. reported on improved patient comfort through use of music [33]. Some of our patients (Figure 1) listen to self-selected music prior to and during the procedure. All patients in our program receive a minimum of four coaching sessions in the first seven days following extubation.

Within our cohort, more invasive interventions such as balloon dilatation and argon photocoagulation were associated with a minimally increased complication rate. Previous reports evaluating balloon dilatation for bronchial stenosis following transplantation concur that the procedure is safe, reporting only on a single minor bleeding episode [34,35]. No data regarding argon photocoagulation has been previously published.

In assessing complication rates dependent upon clinician experience, we observed no significant differences. Comparisons here are, however, difficult, as high-risk procedures were more likely to be performed by skilled examiners. Other studies, retrospectively examining the effect of experience on complication rates amongst clinicians learning bronchoscopy, reported equivalent rates (2.1% versus 1.6%) and demonstrated the highest rates for those in their first trimester [15,36]. Even in inexperienced hands, bronchoscopy was shown to be a safe procedure with an acceptable complication rate. It does, however, highlight the importance of risk-stratifying procedures beforehand, to ensure that examiners of sufficient experience perform the investigation.

Outpatient bronchoscopy in post transplantation care reduces health care costs. In addition, it may also improve quality of life due to lower hospital admission rates.

Limitations of our study include the retrospective design and its realization as a single centre trial. However, due to high follow up rates and high numbers of performed interventions these limitations are to be disregarded.

## Conclusions

Our study verifies both the safety and efficacy of outpatient bronchoscopy in the follow-up management of LTx recipients. Delayed complications occurring beyond the post-procedure observation period appear rare and the procedure demonstrated no associated mortality. The main complications were bleeding and pneumothorax, all of which were managed entirely conservatively. Diagnostic samples obtained from BAL and TBB afforded adequate analysis. For all these results the following conditions should be considered: the patient data were generated in an experienced transplant centre with a well-structured follow up program located in a clinic for pneumology with more than 6,000 bronchoscopic examinations. Sedation was associated with higher complication rates and should be used solely on an as required basis. Experienced clinicians should perform high-risk interventions, such as argon therapy and balloon dilatation. Outpatient bronchoscopy in post transplantation care reduces health care costs and may improve quality of life due to lower hospital admission rates.

The data sets supporting the results of this article are included within the article.

## Abbreviations

BGA: blood gas analysis; BAL: bronchial alveolar lavage; BOS: bronchiolitis obliterans syndrome; CLAD: chronic lung allograft dysfunction; CF: cystic fibrosis; DRG: diagnosis related groups; IFT: immunofluorescence testing; IQR: interquartile range; ISHLT: International Society for Heart and Lung Transplantation; LTx: lung transplantation; SLTx: single lung transplanted; TBB: trans-bronchial biopsy.

## Competing interests

The authors declare that they have no competing interests.

## Authors’ contributions

JR participated in the design of the study, acquisition and analysis of data and writing of the paper. HS participated in statistical analysis and writing of the paper. MG participated in analysis and interpretation of data and in writing the paper. AH has been involved in revising the manuscript critically for important intellectual content. TW has given final approval of the version to be published. GW participated in critical discussion of the manuscript for intellectual content. JG conceived of the study and participated in its design and helped to draft the manuscript. All authors read and approved the final manuscript.
